# The association between metal allergy, total hip arthroplasty, and revision: A case-control study

**DOI:** 10.3109/17453670903487008

**Published:** 2009-12-04

**Authors:** Jacob Pontoppidan Thyssen, Stig Storgaard Jakobsen, Kåre Engkilde, Jeanne Duus Johansen, Kjeld Søballe, Torkil Menné

**Affiliations:** ^1^National Allergy Research Centre, Department of Dermato-allergology, Gentofte Hospital, University of Copenhagen, Copenhagen; ^2^Department of Orthopaedics, Aarhus University Hospital, Aarhus, Denmark

## Abstract

**Background and purpose** It has been speculated that the prevalence of metal allergy may be higher in patients with implant failure. We compared the prevalence and cause of revisions following total hip arthroplasty (THA) in dermatitis patients suspected to have contact allergy and in patients in general with THA. Furthermore, we compared the prevalence of metal allergy in dermatitis patients with and without THA.

**Materials and methods** The Danish Hip Arthroplasty Registry (DHAR) contained detailed information on 90,697 operations. The Gentofte patch-test database contained test results for patients suspected of having allergic contact dermatitis (n = 18,794). Cases (n = 356) were defined as patch-tested dermatitis patients who also had primary THA performed. Two age- and sex-matched controls (n = 712) from the patch-test database were sought for each case.

**Results** The prevalence of revision was similar in cases (12%) and in patients from the DHAR (13%). The prevalence of metal allergy was similar in cases and controls. However, the prevalence of metal allergy was lower in cases who were patch-tested after operation (6%) than in those who were patch-tested before operation (16%) (OR = 2.9; 95% CI = 1–8).

**Interpretation** We found that the risk of surgical revision was not increased in patients with metal allergies and that the risk of metal allergy was not increased in cases who were operated, in comparison to controls. Despite some important study limitations, our observations add to the evidence that the risk of complications in metal allergic patients seems limited.

## Introduction

Metal allergy—defined as contact allergy to chromium, cobalt, or nickel—is prevalent in the general population (Thyssen et al. 2007b). Metal allergy typically develops early in life following prolonged or repeated skin contact with consumer items such as jewelry (Meijer et al. 1995, Hindsen et al. 2005, Thyssen and Maibach 2008, Thyssen et al. 2009a), clothing fasteners (Suneja et al. 2007, Heim and McKean 2009), cell phones (Seishima et al. 2003, Thyssen et al. 2008b), and leather (Geier et al. 2000, Hansen et al. 2006). Upon repeated or prolonged cutaneous exposure, allergic individuals may develop allergic contact dermatitis, an itchy disorder characterized by erythema, papules, and vesicles.

With an increasingly ageing population, total hip arthroplasty (THA) is common. In the 1960s and 1970s, the initial prostheses used for THA were metal-on-metal but these were gradually abandoned, as they resulted in excessive release of cobalt and chromium into the blood, hair, and urine as well as metal sensitization and prosthesis loosening (Coleman et al. 1973, Benson et al. 1975, Elves et al. 1975, Gawkrodger 2003). It remains unclear whether prosthesis loosening at the time was caused by metal allergy or vice versa (Gawkrodger 2003, Jacobs et al. 2009). During the 1970s and 1980s, the use of metal-on-polyethylene prostheses widely replaced the use of metal-on-metal prostheses. Metal allergy is rarely a problem with their use, and allergy does not appear to accompany or cause prosthesis failure (Benson et al. 1975, Rooker and Wilkinson 1980, Waterman and Schrik 1985, Balato et al. 1995).

The popularity of metal-on-metal prostheses is once again increasing, as they have a lower volumetric wear rate, a high fracture toughness combined with the ability to use large femoral heads which may decrease the risk of postoperative instability (Wagner and Wagner 2000, Jacobs et al. 2009). Such prostheses typically consist of a forged, high-carbon cobalt-chromium-molybdenum material but many variations exist (Kim et al. 2008). They produce a greater number of metal particles in nanometer size, which gives a high specific surface area (Jacobs et al. 2009). Some authors have documented elevated serum and urine concentrations of cobalt and chromium (Gleizes et al. 1999, Schaffer et al. 1999, MacDonald et al. 2003, Back et al. 2005, Jacobs et al. 2009). The histopathological response is different from that of conventional metal-on-plastic bearings, as unusual lymphocytic aggregates may be observed in the periprosthetic tissues in patients with metal-on-metal articulations (Davies et al. 2005, Willert et al. 2005, Jacobs et al. 2009). Also, clinically serious complications with aseptic lymphocytic vasculitic associated lesions (ALVAL) and pseudotumors have been reported (Pandit et al. 2008, Mikhael et al. 2009). Finally, T-helper cell 1 response is associated with metal induced reactivity (Hallab et al. 2008). To date, the long-term biological effect of elevated systemic metal concentrations is unknown, although it has been suggested that the prevalence of metal allergy could be higher among patients with implant failure (Hallab et al. 2001, Jacobs et al. 2009).

We had two aims in this study. Firstly, we wanted to investigate a possible association between metal allergy and THA. This was done by comparing the prevalence of metal allergy (defined as contact allergy to nickel, chromium, or cobalt) in patch-tested dermatitis patients with THA (cases) and in patch-tested dermatitis patients (controls) without any known THA. Secondly, we compared the prevalence (and cause) of revisions following THA in patch-tested dermatitis patients who had been treated at a tertiary dermatological referral center (cases) with the prevalence of “ordinary” patients who underwent THA but who were not registered in the patch-test database. This comparison was legitimate, since dermatitis patients have a high prevalence of metal allergy and since their immune system is considered to be highly active and to readily express delayed-type hypersensitivity reactions. Furthermore, metal allergies are likely to precede total hip arthroplasty in most patients, as sensitization usually occurs early in life. Thus, if metal allergy increases the risk of revision and prosthesis loosening, different frequencies of these parameters could possibly be identified in the two study groups.

## Materials and methods

### Study population

In Denmark, a unique civil registry (CVR) number is given to all Danish citizens at birth and also to immigrants when they become Danish residents. The CVR number encodes information about sex and date of birth, and is used for administrative purposes. In this study, we used the CVR number in a linkage study between two clinical databases. The first database contained patch-test results from all patients suspected to have allergic contact dermatitis (n = 18,794) between 1979 and 2007 at the Department of Dermato-allergology, Gentofte Hospital, Denmark. The second database, the Danish Hip Arthroplasty Registry (DHAR) at Aarhus University Hospital, contained information on patients with primary THA and revision performed in Denmark between 1996 and 2007. All 45 orthopedic departments in Denmark, including 5 departments in private hospitals, report to the DHAR (Lucht 2000). The data registered, including pre-, peri- and postoperative data, were collected by the operating surgeon using standardized forms. 150 patients in the DHAR were not Danish citizens and were excluded from the analyses. Thus, the DHAR contained detailed information on 90,697 hip arthroplasty operations and revisions based on 71,054 different patients.

We identified 356 CVR numbers that were present in both databases. We had no information about the reason for patch-testing of these patients, but the vast majority were most certainly patch-tested as a result of current dermatitis. Thus, cases (n = 356) were defined as patients with dermatitis who had been patch-tested at Gentofte Hospital between 1979 and 2007 and who also had primary THA (and revision) performed between 1996 and 2007 at participating orthopedic departments. Two matched controls (n = 712) from the patch-test database at Gentofte Hospital were sought for each case. Controls were therefore defined as patients suspected of having allergic contact dermatitis who had been patch-tested at Gentofte Hospital between 1979 and 2007, and who were not registered in the DHAR. Matching variables included age and sex. Thus, for each case, two matching controls of the same sex and with the same age in years were found. Matching was not performed according to the year or the outcome of the patch-test.

### Patch-testing

Patch-testing was performed with the European baseline series using Finn chambers (8 mm; Epitest Ltd, Oy, Finland) on Scanpor tape (Norgesplaster A/S, Vennesla, Norway). Nickel sulfate, cobalt chloride, and potassium dichromate were tested in petrolatum in concentrations of 5%, 1%, and 0.5%, respectively, according to international standards. The patch-tests were applied to the upper back and were occluded for 48 h. Readings were done on day 2, day 3 or day 4, and day 7 according to the recommendations from the International Contact Dermatitis Research Group (Wilkinson et al. 1970). Thus, homogeneous redness and infiltration in the entire test area was scored as a 1+ reaction. Homogeneous redness, infiltration, and vesicles in the test area were scored as a 2+ reaction, and homogeneous redness, infiltration, and coalescing vesicles in the test area were scored as a 3+ reaction. A 1+, 2+, or 3+ reading was interpreted as a positive response. An irritant response, a doubtful, or a negative reading was interpreted as a negative response.

### Statistics

Comparisons between independent variables were made using the chi-square test. Fisher's exact test was used when the expected frequency in one of the cells was lower than 5. The statistical significance level was set at 0.05. The prevalence of metal, nickel, chromium, and cobalt allergy in cases and controls was compared using conditional logistic regression analysis to take the matching variables (sex and age) into account. Associations were expressed as odds ratios (ORs) with 95% confidence intervals (CIs). Data were analyzed using SPSS for Windows, release 15.0.

## Results

356 patients (defined as cases) were registered in the patch-test database (0.5%) and also in the DHAR. In 64 of the cases (18%), the earliest date was registered in the DHAR (i.e. THA was performed before patch-testing) whereas in 292 cases (82%), the earliest date was registered in the patch-test database (i.e. patch-testing was performed before total hip arthroplasty).

Patient characteristics were generally similar among cases and patients from the DHAR. However, the proportion of female patients was higher among cases (67% versus 58%) (Table 1), which was to be expected as the proportion of women is generally high in Danish patch-test populations (Thyssen et al. 2008a). Also, cases belonged more often to the oldest age group (> 80 years) when compared to patients from the DHAR. This could be attributed to the longer sampling period for dermatitis patients, as the patch database was initiated in 1979 whereas the DHAR only dates from 1996. The prevalence of revision was comparable in cases and patients from the DHAR, as 12% and 13% of patients, respectively, underwent revision between 1996 and 2007 (Table 1). Finally, similar results were found when data were stratified into 3 groups based on the femoral head and acetabular liner materials inserted: (1) ”metal-on-metal”, (2) ”metal-on-polyethylene”, and (3) ”ceramic-on-ceramic” or ”ceramic-on-polyethylene”. The high proportion of missing data was explained by insufficient registration of liner materials in the DHAR, as this variable was introduced in recent years.

**Table 1.** Characteristics of patients undergoing primary total hip replacement and revision between 1995 and 2007

	DHAR ^a^(n=70,698) % (n)	Cases ^b^ (n=356) % (n)	OR (95% CI) ^c^	p-value ^c^
Gender						
Female	58.8	(41, 136)	67	(238)	0.68 (0.55–0.85)	0.001
Age-groups						
10–49	5.8	(4,145)	1.1	(4)	5.45 (2.03–14.61)	0.001
50–59	13	(9,442)	4.5	(16)	3.26 (1.97–5.38)	
60–69	28	(20,060)	23	(81)	1.34 (1.04–1.71)	
70–79	35	(24,821)	32	(113)	1.15 (0.92–1.44)	
>80	18	(12,586)	40	(142)	0.32 (0.26–0.40)	
Primary diagnosis						
Primary osteoarthritis	76	(53,944)	70	(249)	1.35 (1.08–1.70)	0.05
Sequelae of trauma	14	(10,183)	19	(68)	0.71 (0.54–0.92)	
Avascular necrosis	2.4	(1,726)	3.4	(12)	0.71 (0.40–1.27)	
Rheumatoid arthritis	2.1	(1,487)	3.1	(11)	0.67 (0.37–1.22)	
Paediatric conditions	3.4	(2,385)	2.5	(9)	1.34 (0.69–2.60)	
Other diagnoses	1.9	(1,329)	2.0	(7)	0.95 (0.45–2.01)	
Cause of revision						
Aseptic loosening	7.4	(5,284)	7.9	(28)	0.94 (0.64–1.39)	0.2
Osteolysis	0.2	(133)	0.3	(1)	0.67 (0.09–4.77)	
Deep infection	1.3	(908)	1.1	(4)	1.14 (0.42–3.06)	
Fracture or luxation	2.8	(1,986)	1.1	(4)	2.53 (0.94–6.79)	
Other	1.3	(925)	0.6	(2)	2.33 (0.58–9.39)	
Femoral head material						
Metal	76	(53,618)	82	(290)	0.70 (0.54–0.91)	0.2
Ceramics	16	(11,292)	13	(46)	1.27 (0.93–1.74)	
Not exchanged (at revision)	0.004	(29)	0	–		
Other or missing	8.6	(6 115)	5.6	(20)	1.58 (1.01–2.49)	
Acetabular liner material						
Polyethylene	40	(28,425)	31	(111)	1.47 (1.18–1.84)	0.3
Ceramics	2.6	(1,862)	0.8	(3)	3.17 (1.02–9.87)	
Metal	1.6	(1,132)	1.4	(5)	1.14 (0.47–2.75)	
Other or missing ^d^	56	(39,635)	67	(237)	0.63 (0.51–0.79)	
Articulations						
Metal-on-polyethylene	28	(19,933)	23	(83)	1.28 (1.00–1.64)	0.5
Ceramic-on-ceramic or						
ceramic-on-polyethylene	12	(8,211)	7.0	(25)	1.73 (1.15–2.60)	
Metal-on-metal	1.1	(748)	1.1	(4)	0.94 (0.35–2.52)	
Missing ^b^	59	(42,161)	68	(244)	0.67 (0.54–0.84)	

^a^ DHAR: Patients from the Danish Hip Arthroplasty Register (DHAR) 1996–2007. ^b^ Cases, defined as dermatitis patients from the DHAR who were also examined for metal allergy at Gentofte Hospital. ^c^ Comparison of patients from the Danish Hip Arthroplasty Registry (DHAR) and patients from the DHAR who were also examined for metal allergy (Chi-square test). Fisher's exact test was used when the expected frequency in one of the cells was lower than 5. ^d^ The proportion of missing data was high, as the variable was introduced in recent years. OR: odds ratio. CI: confidence interval.

The prevalence of metal allergy including nickel allergy, cobalt allergy, and chromium allergy was similar among cases and controls (Table 2). When stratified by sex, almost identical trends were identified except for cobalt allergy where the prevalence was 5.9% in female cases and 2.5% in female controls (p = 0.3) (data not shown). The prevalence of metal allergy was similar in cases with and without revision of their arthroplasty (Table 3). The prevalence of metal allergy was lower in 64 cases who were patch-tested after operation than in those who were patch-tested before operation (Table 4). This difference was mainly caused by low prevalences of nickel and cobalt allergy in cases who were patch-tested after their operation. The age distribution in patients who were operated before and after patch-testing was similar. Furthermore, the prevalence of revision was similar in patients who were operated prior to patch-testing and in patients operated after patch-testing.

**Table 2.** The prevalence of metal allergies in cases (n = 356) and matched controls (n = 712) from the patch-test database at Gentofte Hospital, Denmark

	Cases (n = 356) % (n)	Controls (n = 712) % (n)	p-value ^a^
Metal allergy ^b^	14 (51)	14 (97)	1.0
Nickel allergy	10 (36)	9.8 (70)	0.8
Cobalt allergy	5.3 (19)	5.2 (37)	0.5
Chromium allergy	3.1 (11)	3.9 (28)	0.4

^a^ Comparison of cases and controls matched by sex and by age in years (conditional logistic regression analysis). ^b^ Metal allergy was defined as a positive patch-test reaction to nickel, cobalt or chromium.

**Table 3.** The prevalence of metal allergies in cases (n = 356) from the patch-test database, stratified by the number of revisions

	Cases operated once (n = 273) % (n)	Cases revised once (n = 70) % (n)	Cases revised two or three times (n = 13) % (n)	OR (95% CI) ^a^	p-value ^a^
Metal allergy ^b^	16 (43)	11 (8)	0	1.38 (0.68–2.80)	0.4
Nickel allergy	11 (29)	10 (7)	0	1.06 (0.48–2.32)	0.9
Cobalt allergy	5.5 (15)	5.7 (4)	0	0.96 (0.32–2.81)	1.0
Chromium allergy	2.9 (8)	4.3 (3)	0	0.68 (0.18–2.51)	0.7

^a^ Comparison of cases without revision and those with one revision (Chi-squared test). Fisher's exact test was used when the expected frequency in one of the cells was lower than 5. ^b^ Metal allergy was defined as a positive patch-test reaction to nickel, cobalt, or chromium. OR: odds ratio. CI: confidence interval.

**Table 4.** The prevalence of metal allergies and the type of material inserted during hip operation in patients who were patch-tested prior to the operation (n = 292) and in patients who were patch-tested after the operation (n = 64)

	Cases patch-tested			
	before operation (n = 292) % (n)	after operation (n = 64) % (n)	OR (95% CI) ^a^	p-value ^a^
Metal allergy ^b^	16 (47)	6.3 (4)	2.87 (1.00–8.30)	0.4
Nickel allergy	11 (33)	4.7 (3)	2.59 (0.77–8.73)	0.1
Cobalt allergy	6.5 (19)	0 (0)	–	0.03
Chromium allergy	3.1 (9)	3.1 (2)	0.98 (0.21–4.67)	1.0
Cause of revision				
Aseptic loosening	7.5 (22)	9.4 (6)	0.79 (0.31–2.03)	0.2
Osteolysis	0	1.5 (1)	–	
Deep infection	1.0 (3)	1.5 (1)	0.65 (0.07–6.39)	
Fracture or luxation	1.3 (4)	0	–	
Other	0.3 (1)	0	–	
Femoral head material				
Metal	81 (236)	84 (54)	0.78 (0.37–1.63)	0.6
Ceramics	13 (38)	13 (8)	1.05 (0.46–2.37)	
Not exchanged (at revision)	0	0	–	
Other or missing	6.2 (18)	3.1 (2)	2.04 (0.46-9.01)	
Acetabular liner material				
Polyethylene	35 (103)	13 (8)	3.81 (1.75–8.31)	0.001
Metal	1.7 (5)	0	–	
Ceramics	1.0 (3)	0	–	
Other or missing	62 (162)	88 (56)	0.18 (0.08–0.39)	

^a^ Comparison of cases and controls (Chi-squared test). Fisher's exact test was used when the expected frequency in one of the cells was lower than 5. ^b^ Metal allergy was defined as a positive patch-test reaction to nickel, cobalt or chromium. OR: odds ratio. CI: confidence interval.

The use of various commercial femoral head components was investigated (data not shown). Apparently, Lubinus SP II and Taberloc stems were used more frequently in cases than in patients from the DHAR (3% vs. 12% (p < 0.001) and 9.3% vs. 2.3% (p < 0.001), respectively) whereas Exeter stems were more prevalent in patients from the DHAR than in cases (18% vs. 4% (p < 0.001)). Lubinus and Universal Ringlocs were used more frequently as commercial liner components in cases than in patients from the DHAR (33% vs. 11% (p < 0.001) and 12% vs. 6.1% (p < 0.001), respectively). No statistically significant differences were identified regarding the use of acetabular liner and femoral head materials between patients tested before and after operation. Also, the prevalence of metal allergy in cases and controls with various commercial femoral head and acetabular liner components was similar to the overall prevalence of metal allergy. Finally, the prevalence of revisions was not associated with any specific components.

## Discussion

We found that the use of metal-on-metal prostheses and the prevalence of revisions and its causes following THA was similar in patch-tested dermatitis patients and “ordinary” patients from the DHAR (Table 1). The Department of Dermatology at Gentofte Hospital is a tertiary referral center where patch-testing of patients with moderate-to-severe allergic and eczematous disease is performed. Referred patients generally have increased delayed-type hypersensitivity immune responses upon cutaneous exposure to contact allergens compared to healthy individuals.

Accordingly, the prevalence of metal allergy was markedly higher in cases (and controls) than in subjects from the general population in Denmark (Nielsen and Menne 1992, Thyssen et al. 2009b). Metal allergy typically develops early in life following cutaneous exposure, and is therefore likely to precede THA in most patients (Thyssen et al. 2007a). If subjects with metal allergy have an increased risk of complications following THA, e.g. aseptic loosening or reoperations, one would expect the prevalence to be higher in dermatitis patients (who had a high prevalence of metal allergy) than in “ordinary” patients from the DHAR (who were suspected of having a prevalence of metal allergy that was comparable to the prevalence in the general population). However, our study results indicate that dermatitis patients generally do not have an increased risk of complications following THA. The patch-test follow-up period following THA in this study was limited to 1–12 years. Thus, a longer period would possibly have revealed further complications. However, as the year of first operation and the year of patch-testing were equally distributed over the study years among cases, the follow-up time was reasonably long for most patients. Reed et al. published their clinical experience from 22 patients who underwent patch-testing before metal device implantation due to a history of contact allergy (Reed et al. 2008). The authors concluded that patch-testing was helpful in guiding the choice of device selected.

A weakness of our study was an insufficient registration regarding various combinations of femoral heads and acetabular liner materials (i.e. ”metal-on-metal”, ”metal-on-polyethylene”, and ”ceramic-on-ceramic” or ”ceramic-on-polyethylene” prostheses) (Table 1). Thus, the study cannot confirm or exclude an association between metal allergy and second generation metal-on-metal prostheses in the entire study material, but only in a sub-sample. It is therefore possible that in patients with missing data (about 60%), an association between second-generation metal-on-metal prostheses and metal allergy, revision, or aseptic loosening might be found. The use of metal femoral stems was significantly higher in cases than in patients from the DHAR (82% vs. 76%). This finding may reflect the fact that ceramic bearings were mainly used in the 1990s and thus that few were present in both databases. Also, it could simply be a result of random error. However, it cannot be completely ruled out that an association exists because of greater metal exposure in patients receiving a metal head than in patients receiving a ceramic head. Our main finding, that metal allergy was not associated with revision or aseptic loosening, was supported by a sub-investigation among cases, which showed that the prevalence of metal allergy was similar in patients who had had no revision performed and in patients who had had one or more revisions (Table 3). However, one should be aware that the study size was small and that a higher number of patients could increase the validity of this finding. Finally, the overall use of various commercial femoral heads and acetabular components was similar in patients from the DHAR and in cases. The prevalence of metal allergy and the proportion of revisions were independent of the commercial subtype of femoral heads and acetabular components.

The prevalence of nickel allergy, cobalt allergy, and chromium allergy was similar in 356 patch-tested dermatitis patients with THA and in 712 patch-tested dermatitis patients without known THA (Table 2). The number of cases was relatively small, which could hide even small differences in the prevalence of metal allergy between cases and controls. However, previous linkage studies with fewer cases have demonstrated inverse associations between contact allergy and prevalent disorders such as diabetes (Engkilde et al. 2006) and inflammatory bowel disease (Engkilde et al. 2007). Furthermore, the prevalence of metal allergy in cases and in controls was similar, and was also the same as that of metal allergies registered in patients from private dermatology practice (Thyssen et al. 2009c). Thus, these findings indicate that THA does not lead to higher prevalences of metal allergy. It is interesting that the prevalence of metal allergy (caused by low prevalences of nickel and cobalt allergy) was statistically significantly lower in 64 patients who were patch-tested after total hip arthroplasty than in 292 patients patch-tested before operation. This finding may be the result of random error. Also, the study sample was very small, which reduces validity. However, it is known that tolerance rather than hypersensitivity may develop in some individuals following systemic exposure to an allergen from e.g. dental braces or drinking water (Van et al. 1991, Smith-Sivertsen et al. 1999, Mortz et al. 2002). Whether or not tolerance could explain the difference between the two groups is unknown. We had no knowledge about the indication for patch-testing; however, the vast majority of patients were patch-tested due to dermatitis and not as a result of surgical complications.

We investigated the overall association between contact allergy to selected metals (nickel, cobalt, and chromium) and total hip arthroplasty but were not able to specifically investigate an association with second-generation metal-on-metal prostheses. We found that the risk of surgical revision was not increased in patch-tested dermatitis patients with metal allergies in comparison to “ordinary” THA patients who were not registered in the patch-test database. Also, we found that the prevalence of metal allergy was not increased in patch-tested dermatitis patients who underwent THA in comparison to patch-tested dermatitis patients who were not operated. When interpreting these results, one should bear in mind that the study method had several limitations, such as small sample size and a case-control study design. Furthermore, although the overall prevalence appeared to be similar between the study groups, delayed-type hypersensitivity reactions would undoubtedly develop following exposure to metal implants in a few selected individuals (Davies et al. 2005, Willert et al. 2005). Furthermore, two prospective studies have found an increased incidence of metal allergy in patients with failed implants (Hallab et al. 2001, Thomas et al. 2009) whereas 2 prospective studies with unselected groups did not find any increase in allergy (Duchna et al. 1998, Schuh et al. 2008). Despite some important limitations of our study design, our findings add to the evidence that the risk of complications in metal allergic patients appears limited.
